# Accurate and reliable quantification of total microalgal fuel potential as fatty acid methyl esters by *in situ* transesterification

**DOI:** 10.1007/s00216-012-5814-0

**Published:** 2012-02-18

**Authors:** Lieve M. L. Laurens, Matthew Quinn, Stefanie Van Wychen, David W. Templeton, Edward J. Wolfrum

**Affiliations:** National Renewable Energy Laboratory, National Bioenergy Center, 1617 Cole Blvd, Golden, CO 80401 USA

**Keywords:** Fatty acids, Fuels, Catalysts, GC, Bioanalytical methods, Algae

## Abstract

In the context of algal biofuels, lipids, or better aliphatic chains of the fatty acids, are perhaps the most important constituents of algal biomass. Accurate quantification of lipids and their respective fuel yield is crucial for comparison of algal strains and growth conditions and for process monitoring. As an alternative to traditional solvent-based lipid extraction procedures, we have developed a robust whole-biomass *in situ* transesterification procedure for quantification of algal lipids (as fatty acid methyl esters, FAMEs) that (a) can be carried out on a small scale (using 4–7 mg of biomass), (b) is applicable to a range of different species, (c) consists of a single-step reaction, (d) is robust over a range of different temperature and time combinations, and (e) tolerant to at least 50% water in the biomass. Unlike gravimetric lipid quantification, which can over- or underestimate the lipid content, whole biomass transesterification reflects the true potential fuel yield of algal biomass. We report here on the comparison of the yield of FAMEs by using different catalysts and catalyst combinations, with the acid catalyst HCl providing a consistently high level of conversion of fatty acids with a precision of 1.9% relative standard deviation. We investigate the influence of reaction time, temperature, and biomass water content on the measured FAME content and profile for 4 different samples of algae (replete and deplete *Chlorella vulgaris*, replete *Phaeodactylum tricornutum*, and replete *Nannochloropsis* sp.). We conclude by demonstrating a full mass balance closure of all fatty acids around a traditional lipid extraction process.

**Electronic supplementary material:**

The online version of this article (doi:10.1007/s00216-012-5814-0) contains supplementary material, which is available to authorized users.

**Figure:**
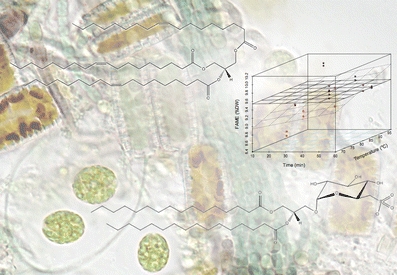
Accurate quantification of different types of lipids in algal biomass using a comprehensive study of reaction parameters.

## Introduction

Algae have significant potential to contribute to the biofuels feedstock pool of the future because of their high biomass productivity and potentially high lipid content of the algal biomass, though many challenges are associated with rendering the production process economical [1, 2]. The current state-of-the-art techno-economic process model demonstrates that the lipid content of the algae has the largest influence on the ultimate cost of the produced biofuel [3]. It is thus important to have robust analytical procedures in place to determine the lipid content as fuel yield.

Even though high lipid productivities are often reported for algal cultures, there is a wide variety of analytical methodologies associated with the reported values, resulting in the potential for greater variability. Therefore, there is a need for robust standardized methods to determine lipid content or biofuel-potential in microalgal biomass. The term “lipids” is a surprisingly vague concept, defined as biochemical compounds not soluble in water but soluble in organic solvents [4]. This definition has been the basis for the quantification of the total lipid fraction of algae as the total quantity of compounds soluble in a chloroform/methanol solvent mixture (based on an original method described by Bligh and Dyer [5] and then improved by Folch et al. [6] and critically reviewed by Iverson [7]). It is clear from the diversity of the published lipid contents of algae and inconsistencies in reported methodology that this loose definition needs to be improved. A recent report by Sheng et al. [8] compares the efficiency of extracting lipids from *Synechocystis* using 15 different solvent mixes and concludes that gravimetric extraction yields are highly dependent on the polarity of the solvents used and the composition of the algal lipids, which is not surprising considering the complex mixture of polar and non-polar lipids that are present in algal biomass.

Since the acyl chains of the lipids will ultimately determine the theoretical fuel potential of algal biomass, a quantification of lipids as the sum of their fatty acid constituents is appropriate. Perhaps a better definition of lipids, in the context of this paper and algal biofuels in general, is “fatty acids and their derivatives”. In this study, we have focused our work on developing a single-step *in situ* transesterification procedure specifically for algal biomass with varying levels and mixtures of algal lipids, omitting the need for an initial lipid extraction. *In situ* transesterification refers to the direct transesterification of lipids in a biomass matrix without prior lipid extraction and offers the advantage of quantifying all fatty acids as fatty acid methyl esters (FAMEs), irrespective of the lipid extraction efficiency [9]. This process is gaining recognition as a lipid measurement procedure for algae [10, 11]; however, a comprehensive study of the reaction yields with different catalysts, tolerance to moisture in the biomass, and a comparison with standard AOAC methods on different algal strains has not been reported previously. In addition, the lack of detailed description of the methodology used in earlier published reports, which hinders the ubiquitous adoption throughout the algae research community.

Transesterification of lipids can be carried out using both acid and base catalysts, or a combination of both. Base catalysis is known to be a much faster reaction compared with acid catalysis, but is also more selective with regards to the types of lipids that are transesterified. For example, free fatty acids are notoriously difficult to convert to fatty acid methyl esters with a base catalyst. If an algal biomass sample contains high concentrations of free fatty acids, the overall FAME yield obtained by base-catalyzed *in situ* transesterification may underestimate the actual FAME yield of the biomass due to partial saponification and soap formation [12]. Nagle and Lemke [13] investigated the effect of acid and base catalysts on the conversion of algal oils and concluded that acid catalysts resulted in consistently higher yields.

Tetramethylguanidine (TMG) is a base catalyst that has been used as a special type of catalyst that reportedly is less sensitive to the presence of free fatty acids. Therefore, TMG appears suitable for performing *in situ* transesterification and has been used successfully on oilseeds [14]. However, this method has not been applied to algae and was included in our studies because of its reported simplicity, fast reaction rate, and potential tolerance to high levels of moisture in the biomass.

Several standard procedures for quantification of lipids and fatty acids are listed by AOAC International (Association of Analytical Communities) and are routinely used in the food and agricultural industries (e.g., AOAC 922.06, 989.05, 991.39). No methodology specific to algal biomass has been reported by AOAC, but some of the listed methods have been applied to algal biomass [10, 11]. The 989.05 method for fat analysis in milk includes a NH_4_OH pretreatment step to dissolve the major milk protein casein followed by an ether extraction of the residue and a gravimetric determination of the fat content. We included modifications of these methods in our study in view of the fact that algae are known to be protein-rich and a protein hydrolysis step could liberate additional FAMEs from a complex protein matrix. Similarly, the 922.06 method for flour includes a concentrated HCl-hydrolysis step prior to transesterification to hydrolyze the carbohydrates and release FAMEs. Because of the reported rigid, carbohydrate-rich cell walls in algae. The AOAC 991.39 method was published as an effective transesterification method for quantification of FAMEs in fish oil and consists of a two-step catalyzed reaction using NaOMe followed by BF_3_. Modifications of this method are referred to as NaOMe/BF_3_ methods throughout this manuscript and are compared to our single-step acid catalysis method.

The ability of acid to catalyze the esterification of all fatty acids (free or linked) has led to a widespread use of acid catalysis, even though heating is required and the reaction time needed is longer when compared to base catalysis. A variety of acids have been used in the past for transesterification of lipids and free fatty acids, but methanolic hydrogen chloride (referred to as HCl/MeOH) is, according to some authors, the best general purpose esterifying agent and the most widely mentioned catalyst being used for *in situ* procedures [9, 15, 16]. We have modified the Lepage and Roy [16] HCl-catalyzed procedure to suit algal biomass hydrolysis and transesterification in a simple, single-step reaction. Ehimen et al. [17] studied the variables affecting the *in situ* transesterification of microalgal lipids. The authors investigated the effect of alcohol volume, temperature, and reaction time and moisture on transesterification and reported a requirement for greater catalyst loading, alcohol volume (due to elevated methanol consumption), and a high sensitivity to water in the biomass. Since the objective of Ehimen et al. [17] was to define relevant conditions for an industrial-scale process and our emphasis is on developing a small-scale analytical procedure, we are less concerned about catalyst loading and methanol requirement. The aim of our study was to understand the tolerance of the acid-catalyzed reaction to water in order to establish moisture content limits for the analyzed algal biomass. Furthermore, it was important that we minimize degradation due to oxidation of the fatty acids and be able to account and report on the total fatty acid content and profile, both of which are key factors in downstream fuel quality.

Another parameter in algal lipid analysis is the application of the method to small quantities of biomass. Lipid analysis in algae often has to contend with quantities of biomass of 20 mg dry weight (DW) or less, as is typically generated in the lab. Biomass concentrations of typical algal cultures are low (<1 g L^−1^), thus only small quantities of biomass samples can be generated in laboratory-scale shake flask cultures. Setting a minimum sample size at the milligram level allows for replicates and multiple time points using small volumes of algal cultures. Therefore, adaptation of a procedure to small amounts of biomass was a priority in this work. There have been reports in the literature recently that list small-scale adaptations of the *in situ* transesterification as the preferred method for lipid quantification in microalgae, in fact a sub-microscale *in situ* procedure was recently reported by Bigelow et al. [10]. Because of concerns about the accuracy of weighing out biomass samples less than 1 mg in most laboratories, we have focused our work on developing a procedure to work on between 4 and 15 mg biomass, equivalent to 50–100 mL harvested and lyophilized culture per replicate measurement.

## Materials and methods

### Materials

Lyophilized algal biomass was either obtained from a collaborator or grown in our facilities. *Nannochloropsis* sp. was kindly provided by Dr. Ami Ben-Amotz (Nature Beta Technologies, Israel). For the optimization work, we used this biomass because of its abundance. We have also included this material as an internal reference standard to historically track method reproducibility and instrument behavior between multiple reactions. Unfortunately we do not have much information on the exact conditions that were used to grow this biomass, but the measured low lipid content suggests it was produced under nitrate replete conditions and harvested while the culture was actively growing. Other representative algae that were grown *in house* include a *Chlorella vulgaris* (UTEX395) as a model green alga and *Phaeodactylum tricornutum* (P632) as a model diatom. *C. vulgaris* was grown in a modified Bold’s Basal media (0.17 mM CaCl_2_ **·**2H_2_O, 0.304 mM MgSO_4_ **·**7H_2_O, 0.431 mM K_2_HPO_4_, 1.29 mM KH_2_PO_4_, 0.428 mM NaCl, and 3.22 μM FeCl_3_ **·**6H_2_O and trace metals: 80.6 μM Na_2_EDTA**·**2H_2_O, 0.185 mM H_3_BO_3_, 30.7 μM ZnSO_4_ **·**7H_2_O, 7.28 μM MnCl_2_ **·**4H_2_O, 4.93 μM Na_2_MoO_4_ **·**2H_2_O, 6.29 μM CuSO_4_ **·**5H_2_O, 1.68 μM Co(NO_3_)_2_ **·**6H_2_O, 0.352 mM Na_2_SiO_3_ **·**9H_2_O), with or without 10 mM NaNO_3_ in the media. *C. vulgaris* was grown in either a photobioreactor bag set-up in 30 L volumes with 2.5% CO_2_ sparging and 24 h constant light (400 μmol photons). To increase the lipid content in *C. vulgaris* biomass, we reduced the nitrate concentration to 1 mM in the growth media. This biomass is referred to as “deplete” biomass, as opposed to low lipid containing, “replete” biomass. *P. tricornutum* (P632) was grown in Df/2 media (445 mM NaCl, 23.7 mM MgCl_2_ **·**6H_2_O, 27.1 mM MgSO_4_ **·**7H_2_O, 9.39 mM KCl, 1.36 mM NaBr, 36.2 μM NaH_2_PO_4_ **·**H_2_O, 10.0 mM NaNO_3_, 8.81 mM CaCl_2_ **·**2H_2_O; trace metals, 80.6 μM Na_2_EDTA**·**2H_2_O, 0.185 mM H_3_BO_3_, 30.7 μM ZnSO_4_ **·**7H_2_O, 7.28 μM MnCl_2_ **·**4H_2_O, 4.93 μM Na_2_MoO_4_ **·**2H_2_O, 6.29 μM CuSO_4_ **·**5H_2_O, 1.68 μM Co(NO_3_)_2_ **·**6H_2_O, 0.352 mM Na_2_SiO_3_ **·**9H_2_O). *P. tricornutum* was grown in an open pond (250 L) with 5% CO_2_ sparging and natural daylight.

### Lipid extraction

#### Soxhlet lipid extraction

For lipid extraction, a Soxhlet-based extraction procedure was modified from Guckert et al. [18] for the isolation of lipids from ground and lyophilized algal biomass. The biomass was weighed out into single thickness cotton cellulose thimbles and covered with a glass fiber filter. Chloroform/methanol (2:1, *v*/*v*) was allowed to reflux over the thimble for 3 or 16 h (depending on the ease of lipid extraction) at a siphon rate of 6–8 times per hour. The extracts were then quantitatively transferred and brought up to a known volume with chloroform/methanol (2:1, *v*/*v*). An aliquot of the known volume was transferred to a separation funnel and a 0.7–0.75% NaCl (aq) solution was mixed with the aliquot at a final ratio of 8:4:3 (chloroform/methanol/NaCl (aq)) to wash out the non-lipid components from the extract, similar to the washing procedure described by Folch et al. [6]. The resulting biphasic mixture was allowed to settle for 12 h. After settling, the lower phase, consisting of the washed lipids, was drained into a pre-weighed round bottom flask and the solvent was removed using vacuum rotary evaporation at 30–35 °C. The flasks were then placed into a 40 °C vacuum oven for further drying. The dried extracts were weighed to determine lipids. In order to quantify the FAMEs in the extracts, an aliquot (~2 mL) was taken before the extracts were washed, solvent evaporated to dryness and transesterified (using the HCl/MeOH protocol) followed by GC analysis as described below.

#### Accelerated solvent extractor (ASE) lipid extraction

Lipids were extracted with a pressurized fluid extraction system (Accelerated Solvent Extractor 200, Dionex, USA; now Thermo-Fisher). Biomass (200 mg) was added to a stainless steel cell with 11 mL capacity, covered with glass fiber filters on both sides of the biomass, and 3 mm glass beads to fill the extra space in the cell, and extracted with either hexane/isopropanol (3:2, *v*/*v*) or chloroform/methanol (2:1, *v*/*v*). The extraction system was set to reach the following temperatures; 40 °C and 100 °C and pressures; 500 psi and 2,000 psi, the extraction was completed with 5 min solvent residence time and 5 extraction cycles for each extraction. Two consecutive extractions were carried out on each biomass sample and combined, to ensure complete extraction. Triplicate sample preparations were run at each condition. The recovered extract was evaporated under a stream of nitrogen (Turbovap) and the gravimetric extract weight was recorded after further drying over night in a vacuum oven at 40 °C.

### *In situ* transesterification

#### HCl/MeOH

The HCl-catalyzed procedure was modified from that of Lepage and Roy [16]. The method was adapted to a small scale on biomass quantities of between 7 and 11 mg of biomass and was carried out on lyophilized cells (dried overnight at 40 °C under vacuum), solubilizing the lipids in 0.2 mL chloroform/methanol (2:1, *v*/*v*), and simultaneously transesterifying the lipids *in situ* with 0.3 mL HCl/MeOH (5%, *v*/*v*) for 1 h at 85 °C in the presence of 250 μg tridecanoic acid methyl ester (C13-FAME) internal standard. We tested this method with and without a chloroform/methanol presoak step. The resulting FAMEs were extracted with 1 mL hexane at room temperature for at least 1 h and a 1:10 dilution of the extract in hexane was quantified by gas chromatography (GC), as described below.

The internal standard was added at the onset of the reaction to correct for the loss of FAME during the reaction and to correct for incomplete hexane extraction efficiency. The final quantified FAME concentrations obtained after GC analysis were normalized for the C13-FAME concentration (250 μg mL^−1^ hexane) in the original reaction. We measured 89.5 ± 3.5% recovery of total FAMEs and 85.1 ± 0.9% recovery of the internal standard in the single hexane extraction. These recoveries are comparable to our historical C13-FAME recoveries of 87.64 ± 3.04% over 125 individual assays. When we extracted FAMEs with multiple consecutive hexane extractions, we noticed an increase in the recovery of the FAMEs. However, because the inclusion of the C13 internal standard to correct for FAME extraction efficiency, the accuracy of FAME quantification in algal biomass with multiple or single hexane extractions did not change significantly (data not shown). This observation confirms the applicability of a C13-FAME internal standard as a correction factor for incomplete extraction in our single-step procedure and to account for dilution variability during sample preparation. The basic method was modified and optimized with regard to reaction conditions and extraction time and temperature as shown in the “Results” section.

#### NaOMe/BF_3_/AOAC 991.39

The standard AOAC method 991.39 was carried out according to the standard published procedure with the following modifications; 7–11 mg of biomass in the presence of 250 μg C13-FAME internal standard was used for the micro-scale procedure adaptation and both 0.3 mL 0.5 M NaOMe and 0.2 mL 14% BF_3_ (*w*/*v*) in methanol were added simultaneously to the biomass sample for the single-step procedure and the incubation time at 100 °C was varied between 15 and 35 min. The resulting FAMEs were extracted after cooling to 40 °C with 1 mL hexane, followed by the addition of 0.2 mL saturated NaCl to wash the hexane extract, and quantification of the FAMEs by GC.

#### NaOMe

*In situ* transesterification by NaOMe alone was carried out on 7–11 mg of biomass in the presence of 250 μg of C13-FAME internal standard, by addition of 0.5 mL 0.5 M NaOMe and heating at 100 °C for 35 min. The resulting FAMEs were extracted with 1 mL hexane, followed by a saturated NaCl wash and quantification by GC.

#### Tetramethyl guanidine

Catalysis by TMG was carried out using a procedure modified from Schuchardt and Lopes [14]. The scaled down procedure included the addition of 200 μL of TMG/MeOH (1:4, *v*/*v*) to 7–11 mg of biomass, in the presence of 250 μg of C13-FAME internal standard. After incubation at 85 °C for 5 min, 200 μL saturated aqueous solution of NaCl was added and the resulting FAMEs were extracted with 300 μL hexane. The reaction conditions were varied by modifying the temperature and time of the reaction between 20 °C and 90 °C and 5 to 60 min.

#### AOAC 922.06/989.05

Method 922.06 was modified from the standard published procedure and carried out as follows on 7–14 mg of biomass in the presence of 250 μg of C13-FAME internal standard: concentrated hydrochloric acid (200 μL) was added and samples were heated for 5 min at 100 °C on a heating block. After cooling, 200 μL of 2:1 chloroform/methanol was added with an additional 300 μL of HCl/MeOH, per the HCl/MeOH transesterification procedure described above.

Method 989.05 was modified and carried out as follows on 7–14 mg of sample, followed by addition of 300 μL concentrated ammonium hydroxide and incubation at room temperature for 30 min, 250 μg of C13-FAME internal standard, and 1.5 mL of chloroform/methanol (2:1, *v*/*v*) was added. The vials were capped, vortexed, and heated for 10 min at 85 °C and allowed to cool. Hexane (2 mL) was then added, the vials were capped and vortexed allowing the hexane layer to settle for 15 min. The dried hexane extract residue was then transesterified in the presence of 200 μL of chloroform/methanol (2:1, *v*/*v*) was added with an additional 300 μL of HCl/MeOH, per the HCl/MeOH transesterification procedure described above.

#### Pure lipid transesterification

Pure lipids were purchased from Sigma-Aldrich; Palmitic Acid (Cat #R-420160, lot: LB40764, purity: 99.6%), 1-stearoyl-rac-glycerol (Cat #M2015-100MG, lot: 067K5207; purity, 99.9%), eicosanoic acid (Cat #R-420200, lot: LB48664; purity, 99.8%), triheptadecanoate (Cat #T2151-1G, lot: 078K5207; purity, 98.9%), dilaurin (Cat #D9758-100MG, lot: 107K1587; purity, 99.5%), and phosphatidylcholine (Cat #P3556-1G, lot: 118K5206). A total of 5 mg of each individual lipid was treated as a sample in the transesterification reactions, dried overnight under vacuum, and subjected to transesterification by both the HCl/MeOH and the NaOMe/BF_3_ method (as described above), in the presence of 2 mg of C13-FAME internal standard. The resulting FAMEs were extracted in 1 mL hexane and a 1:100 dilution of this extract was analyzed by GC. The FAME yields were calculated as a fraction of the original weights after normalization to the internal standard C13-FAME.

#### Water tolerance experiments

To create biomass samples with a controlled amount of moisture, oven-dried algal biomass samples were spiked with increasing amounts of nanopure water to achieve the following levels: 10%, 30%, 50%, and 80% moisture. To 7–10 mg dried biomass, we added approximately 1, 3, 10, and 40 mg water; the actual water requirement was calculated based on the individual biomass weights.

#### Fatty acid mass balance experiment

To investigate the fatty acid extraction efficiency around an extraction procedure, algal biomass from *Nannochloropsis* sp., *C. vulgaris* deplete, and *C. vulgaris* replete were extracted by Soxhlet (as described above) and samples from the lipid extract (4–7 mg lipids) and residual biomass (10–12 mg) were used for transesterification using the same HCl/MeOH procedure described above for *in situ* transesterification.

### Gas chromatography

FAMEs were extracted from the various transesterification treatments with hexane at room temperature for at least 1 h (but less than 4 h) and analyzed by GC-FID (Agilent 6890N, HP 5-MS column (Agilent, USA), 30 m 0.25 mm ID and 0.25 μm FT, temperature program 70–300 °C at 20 °C min^−1^, plateau for 1 min at 230 °C, at a 1.5 mL min^−1^ He constant carrier gas flow). Quantification of the FAMEs was based on integration of individual fatty acid peaks in the chromatograms and quantified using a 5-point calibration curve (0.5–2 mg mL^−1^) prepared with standards containing 14 even carbon chain FAMEs (C8–C24, SIGMA cat #18918) and 5 odd carbon chain FAMEs (C13–C21, SIGMA cat #1896). The individual FAME concentrations, quantified by GC software (Chemstation B.04.02, Agilent, USA), were normalized against the internal standard concentration of 250 μg C13-FAME. The instrument detection limit (IDL) of this method and calibration was 2.5 μg mL^−1^ per individual FAME and the reproducibility between replicate injections was <5% RSD (data not shown). The limit of detection (LOD) of fatty acids in a biomass sample was 0.025% of the biomass, assuming 7–10 mg of biomass and extraction of the resulting FAMEs in 1 mL hexane. The yields were calculated for each treatment or reaction condition as the sum of the even-numbered FAME concentrations and the profile was studied for differences in the contribution of individual fatty acids to the total FAME yield.

#### Statistical analysis of the results

Unless stated otherwise, all statistical analyses of the results were performed using the statistical program R version 2.13.1 [19], and Electronic Supplementary Material contains the raw data and scripts used for the analyses (Electronic Supplementary Material Text S1, Table S1, Table S2, and Table S3). The significance level used for all conclusions was *p* < 0.05 and was derived from an analysis of variance (ANOVA) followed by a Tukey’s HSD test.

#### Experimental design

To test the robustness of the HCl/MeOH procedure across temperature and time intervals and across different species, we ran designed experiments on four algal biomass samples (replete and deplete *C. vulgaris*, replete *P. tricornutum*, and replete *Nannochloropsis* sp.). We used Design-Expert version 8.0.4 (Stat-Ease, Inc, Minneapolis, MN) to design the experiments and evaluate the results. We used *p* = 0.05 as the test of significance. In order to find optimal reaction conditions, we initially ran a 2^2^ factorial design with a center point testing reaction time and temperature, covering the range 30–90 min and 50–100 °C and we ran four replicates at each condition. We used total FAME yield as the response. This initial experiment was followed up with a central composite response surface design to identify optimal conditions. We covered the range 25–54 min and 75–95 °C for the factorial conditions, 39.5 min and 85 °C for the center point, and used an alpha of 1.41 to determine the axial conditions. We ran duplicates at each factorial and axial condition, plus 5 center point conditions. We again used total FAMEs as the experimental response.

## Results and discussion

### Lipid quantification by solvent extraction

**Fig. 1:**
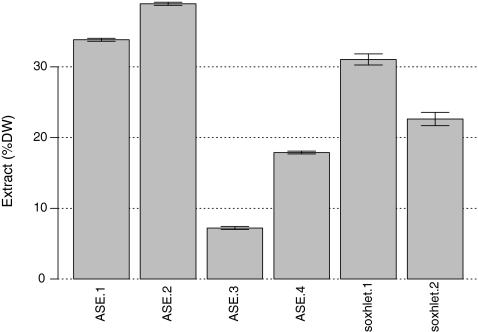
Quantitative extracted lipid yields (as % DW) of *Nannochloropsis* sp. biomass by gravimetric determination after solvent extraction using six different procedures, four of which used an accelerated solvent extractor (ASE) with different temperature and pressure conditions. All data shown are the mean ± SD of triplicate extractions, from L to R: *ASE.1* 40 °C 500 psi with chloroform/methanol (C:M); *ASE.2* 100 °C 2,000 psi C:M; *ASE.3* 40 °C 500 psi with hexane/IPA (HI); *ASE.4* 100 °C 2,000 psi with HI; *Soxhlet.1* 16 h Soxhlet extraction with CM; *Soxhlet.2* 3 h Soxhlet extraction with CM followed by extract washing

To demonstrate the variability of lipid extraction with varying conditions, we extracted the lipids from one biomass sample (*Nannochloropsis* sp.) using six different extraction conditions. The gravimetric yields were found to vary between 7% and 37% DW and are shown in Fig. 1. Total yield of lipid extraction was dependent on the solvent system and lipid extraction parameters used. The pressurized fluid extractor has potential to provide higher throughput extractions of algal biomass; however, we found that the total gravimetric yields are also dependent on the temperature and pressure. Figure 1 illustrates that when the same solvent is used, the extraction efficiency depends on the severity of the extraction conditions. Raising the temperature and the pressure during pressurized fluid extraction increases the gravimetric recovery for both chloroform/methanol (2:1) and hexane/isopropanol (3:2) solvent systems. Using hexane/isopropanol as a solvent reduces the overall extraction yield by ~70% or ~50%. The Soxhlet extractions vary with duration of the extraction and whether or not the extract is subjected to washing prior to weighing. The results illustrate the fact that lipid yields are difficult to quantify using a gravimetric approach.

Lipid fractions resulting from solvent extraction contain non-fuel components (e.g., chlorophyll, pigments, proteins, and carbohydrates that make up part of the glycolipids, e.g., galactose from galactolipids). Fuel potential in our context is defined as the fraction of the lipids composed of fatty acids that are amenable to transesterification. To investigate whether gravimetric lipid extraction yield reflects the fuel potential, we have measured the fuel fraction of extracts, by conversion of fatty acids to FAMEs. For this experiment, we have included representative biomass samples from a low and high lipid containing (replete and deplete respectively) *C. vulgaris* strain. Different lipid types can be converted to fuel to varying degrees, for example triglyceride lipids convert to 100% FAME on a gravimetric basis, since the addition of the methyl group to the fatty acids balances the loss of a glycerol in the hydrolysis step. Due to a larger proportion of the mass associated with the carbohydrate functionality, a glycolipid such as digalactosyl diglyceride converts to 63% FAME, thus the relative composition of the lipids affects the fuel potential of the extracted lipids [13]. Including algal biomass with different lipid compositions presumably will affect the FAME yields of the lipid extracts. We used both a low and high lipid containing biomass sample (assumed to have a low and high concentration of triglycerides respectively) from one strain allowed us to investigate the lipid extraction and conversion efficiency for different lipid composition samples. We found that for deplete *C. vulgaris* biomass the fuel yield of the extract almost matches the weight of the lipid extract (93.3% of the lipids can be converted into FAME). For both replete *C. vulgaris* and *Nannochloropsis* sp., the gravimetric extraction yield far exceed the actual fuel yield; only 30.9% and 51.4% of the extract weight was converted to FAME. This points to further limitations of lipid quantification through extraction and gravimetric recovery, demonstrating that gravimetric recovery may not represent the fuel potential of algal biomass. When we calculated the FAME yield after extraction of lipids for replete and deplete *C. vulgaris* and *Nannochloropsis* sp., we found that lipid extraction followed by quantification of the fuel yield as FAME represents 72%, 49%, and 97.5%, respectively, of the potential fuel yield of the biomass. This reflects both incomplete extraction of lipids and discrepancy between the gravimetric yields and FAME yields of the extracts. Because of these inconsistencies, we have developed an alternative lipid quantification procedure through *in situ* transesterification of the algal biomass.

### *In situ* transesterification and comparison between catalysts

**Fig. 2:**
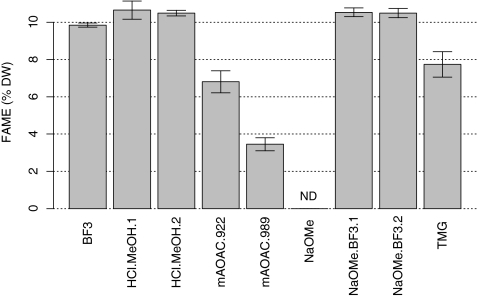
Measured FAME content (% total DW) for *in situ* transesterification of *Nannochloropsis* sp. using nine different conditions (catalysts, or combinations of reaction conditions as described in the text). Each value shown is the average of *N* reactions ± SD. From L to R: *BF*_*3*_ (boron trifluoride) is the single acid catalyst reaction, *HCl.MeOH.1* is the standard reaction as described in the text (*N* = 22), and *HCl.MeOH.2* is the standard reaction omitting a presoaking step with chloroform/MeOH (*N* = 3). *mAOAC.922* and *mAOAC.989* are the respective modified reactions as described in the text. *NaOMe* (sodium methoxide) is the single base catalysis reaction, *NaOMe.BF*_*3*_ *.1* and *NaOMe.BF*_*3*_ *.2* are both modifications of the standard AOAC.991.39 procedure, using the full-scale and the small-scale adaptation respectively (as described in the text), and *TMG* (tetramethylguanidine) is the single base catalyst reaction. *ND* not detected

Different catalysts for *in situ* transesterification are reported in the literature and AOAC procedures. We studied the overall FAME yield obtained with different procedures, including both base and acid catalysts (or a combination of both). Our goal was to find an easy single-step procedure that is robust across different strains and conditions. For this, we modified a published procedure based on HCl as the acid catalyst in a single step reaction [16]. Our modification included the use of a convenient reagent prepared by commercially available concentrated HCl in methanol rather than preparing an anhydrous reagent by adding acetyl chloride to methanol as was described by Lepage and Roy [16]. This modification simplified the procedure for our purposes and has been described by Ichihara [15] to not affect the conversion efficiency of fatty acids. We also included a prior solubilization step of the lipids with chloroform/methanol to aid with access of the reagents to the lipids embedded in the algal cell matrix. To compare our method to an AOAC method previously used for *in situ* FAME quantification in algae (AOAC 991.39, also referred to as NaOMe/BF_3_), we ran both methods simultaneously. In addition, we compared the FAME yields obtained using two more extraction procedures, AOAC 922.06 and AOAC 989.05. The data in Fig. 2 illustrate the FAME yields across different catalysts and an analysis of the variance with Tukey’s HSD test of the results indicates that there was no significant difference between the HCl/MeOH and the standard AOAC 991.39/NaOMe/BF_3_ procedure. Although the original AOAC 991.39 method was designed to use 19 mg of oil, we adapted the method to reduce the overall sample requirement to 4–10 mg. We found that both the full-scale and the small-scale reaction yielded equivalent FAME recoveries (NaOMe/BF_3_.1 and NaOMe/BF_3_.2, respectively, in Fig. 2). We measured the efficiency of FAME conversion using only BF_3_ as the reagent and observed a significant drop in the conversion yield. Furthermore, when using only NaOMe we did not detect any FAME yield at all, indicating that the two stages (NaOMe and BF_3_) are necessary to obtain yields comparable to our HCl procedure (Fig. 2). Similarly, we tested the necessity of presoaking biomass in chloroform/methanol (2:1, *v*/*v*) for the HCl/MeOH procedure and found no significant difference. However, we envisage that the presence of the chloroform/methanol in a presoaking step will help with solubilizing the varying lipid types so we have left this step in our procedure. When we looked at the FAME profiles obtained by both the HCl/MeOH and the small-scale NaOMe/BF_3_ methods we saw no differences between the results from two methods, even when applied to three biomass samples representing high and low lipid containing algae (Table 1).

**Table 1:** Comparison of the FAME profile between the NaOMe/BF_3_ procedure and the HCl/MeOH method for three biomass investigated

	*Nannochloropsis* sp.		*C. vulgaris* replete		*C. vulgaris* deplete	
	NaOMe/BF_3_	HCl/MeOH	NaOMe/BF_3_	HCl/MeOH	NaOMe/BF_3_	HCl/MeOH
C14	4.36 ± 0.02	4.49 ± 0.03	0.11 ± 0.1	0.16 ± 0.02	0.15 ± 0	0.15 ± 0.01
C16:3	1.11 ± 0.01	0.8 ± 0.02	0.38 ± 0.36	0.31 ± 0.31	0.01 ± 0	ND
C16:4	0.97 ± 0	0.93 ± 0.01	0.1 ± 0.17	ND	ND	ND
C16:2	ND	ND	6.19 ± 0.07	6.05 ± 0.11	2.45 ± 0.01	2.45 ± 0.07
C16:1n9	36.28 ± 0.12	35.14 ± 0.18	12.5 ± 0.3	12.34 ± 0.8	8.58 ± 0.11	8.69 ± 0.18
C16:1n11	1.23 ± 0.04	2.03 ± 0.03	ND	ND	ND	ND
C16	20.31 ± 0.05	19.98 ± 0.1	18.44 ± 0.15	18.33 ± 0.33	17.84 ± 0.04	18.04 ± 0.49
C18:2	2.46 ± 0.02	2.32 ± 0.02	15.3 ± 0.15	15.11 ± 0.26	6.57 ± 0.19	6.7 ± 0.09
C18:1n9	3.04 ± 0.01	3.01 ± 0.03	17.26 ± 0.92	18.91 ± 2.11	62.59 ± 0.04	61.9 ± 0.96
C18:3	0.51 ± 0.01	0.5 ± 0.02	26.63 ± 0.53	23.99 ± 1.57	ND	ND
C18	0.4 ± 0.02	0.41 ± 0.02	1.25 ± 0.08	1.18 ± 0.03	1.25 ± 0.02	1.28 ± 0.06
C20:4	4.3 ± 0.03	4.47 ± 0.04	ND	0.01 ± 0.01	0.02 ± 0.01	ND
C20:5	22.39 ± 0.14	23.13 ± 0.24	ND	0.02 ± 0.04	ND	ND
C20	0.36 ± 0.02	ND	ND	ND	ND	ND
C24	0.7 ± 0.07	0.8 ± 0.02	1.08 ± 0.28	0.89 ± 0.15	0.32 ± 0.03	0.35 ± 0.12
Total	10.52 ± 0.02	10.88 ± 0.15	9.8 ± 0.11	10.05 ± 0.22	56.44 ± 0.37	56.54 ± 0.7

Each value is the FAME yield (% DW) of individual fatty acids together with the sum (Total) as the mean ± SD of three replicate measurements

*ND* not detected

Interestingly neither of the two base-catalysis methods (NaOMe and TMG) showed FAME yields comparable to the yields obtained by acid catalysis (Fig. 2). The NaOMe-only reaction showed no detectable FAME yield and the TMG reaction did not match the highest yields observed by acid catalysis. Even after optimizing the reaction conditions over a range of different time and temperature intervals (data not shown), there is still a considerable lack of conversion efficiency compared to the acid catalyzed reaction yields. It is not clear what caused the lack of conversion. Likely explanations are the lack of penetration of the catalyst through the algal cell walls and the presence of free fatty acids leading to a lower efficiency by this base catalyst. Furthermore, the modified AOAC 922.06 and 989.05 procedures showed an overall lower FAME yield compared with the HCl/MeOH or combined base/acid (NaOMe/BF_3_) procedures (Fig. 2). This indicates that these two procedures, although promising with regards to selective pretreatments of the biomass for high protein and carbohydrate containing samples, are not selective (or are perhaps even destructive) to conversion of the fatty acids in algal biomass.

To investigate a potential selectivity towards specific lipid types, we have looked at the FAME conversion efficiency of 6 different lipids, including free fatty acids, tri-, di-, and monoglycerides, and a phospholipid (Table 2). It is worth noting that not all lipids convert to 100% FAME on a weight basis. Theoretical yields are calculated as the ratio of the molecular weight of the respective FAMEs to the molecular weight of the pure lipids presented (MW_FAME_/MW_Lipid_). The data in Table 2 show that only the weight of pure triglycerides can be recovered as the resulting FAMEs (e.g., [3 × 384.5]/849.5 = 100.5%]), a mono-glyceride (1-stearoyl-rac-glycerol) yields only 83.2% FAME (e.g., [1 × 298.3]/358.6 = 83.2%]) and a phospholipid, like phosphatidyl choline, has an even lower FAME yield (64.4%). Table 2 shows that the measured FAME yields correspond to the theoretical yields within 4% (absolute) for both methods. This indicates that neither method exhibits a bias towards specific lipid types. In addition we could not detect a statistically significant difference between the yields obtained with both methods, supporting the statement that both methods are equivalent for the conversion of different lipids.

**Table 2:** FAME yield for individual, commercially available lipids, and the efficiency of conversion with two catalysts

	MW_Lipid_	MW_FAME_	Theoretical yield (%)	HCl/MeOH yield (%)	NaOMe/BF_3_ yield (%)
Palmitic acid	256.4	270.5	105.5	110.05 ± 2.13	103.32 ± 0.44
1-Stearoyl-*rac*-glycerol	358.6	298.3	83.2	85.42 ± 0.76	85.41 ± 3.7
Eicosanoic acid	312.5	324.5	103.8	105.42 ± 0.16	103.13 ± 1.75
Triheptadecanoate	849.4	853.4	100.5	100.5 ± 1.23	98.8 ± 0.32
Dilaurin	456.7	428.7	93.9	93.03 ± 1.5	95.06 ± 2.06
Phosphatidylcholine	NA	NA	NA	64.4 ± 0.8	65.1 ± 0.9

Each lipid was subjected to either HCl/MeOH or the small-scale NaOMe/BF_3_ *in situ* procedures and the recovery of the FAME yield was expressed as % of the original weights of the lipid added to the reaction. For comparison purposes, the molecular weights of the lipids and their corresponding fatty acid methyl esters and the calculated theoretical yields (MW_FAME_/MW_lipid_) are shown

*NA* data on the molecular make up of the lipid was not available

The data shown in Fig. 2 and Table 2 indicate that our HCl/MeOH method is similar to the original and to the small-scale modification of the two-stage NaOMe/BF_3_ procedure. This is a significant finding because HCl is easier to prepare, it is less costly and less caustic than BF_3_, and more stable over longer storage times, thus making the HCl procedure more amenable to wide spread use for algal lipid analysis. We have chosen the acid-catalyzed transesterification method (HCl/MeOH) to proceed based on its yield of FAME, stable FAME profile, and effective conversion of all types of lipids investigated.

### Optimization of HCl/MeOH reaction conditions for *in situ* FAME procedure

**Fig. 3:**
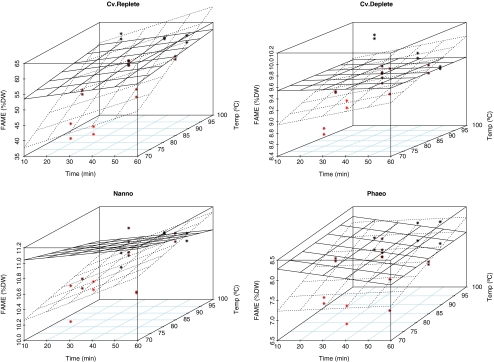
FAME yield (% DW) for HCI:MeOH for varying reaction conditions for *Nannochloropsis* sp., *C. vulgaris* deplete and *C. vulgaris* replete and *Phaeodactylum tricornutum*. Every data point represents a single measurement, the planes represent a linear correlation of all datapoints (*dashed lines*) and datapoints where reaction time ≥60° C and temperature ≥75° C (*solid lines*)

As a measure of the robustness of the procedure, we have performed a detailed analysis of the response of FAME yield over different conditions. A central composite experiment was designed to find optimal reaction conditions over a range of reaction conditions. A full quadratic model was fitted for deplete *Chlorella* and reduced quadratic models (without the interaction term) were used for *Nannochloropsis* sp. and *P. tricornutum* (P632). The deplete *C. vulgaris* model was the only model to show a significant lack of fit. A full interaction model was used for replete *C. vulgaris* (details on model statistics are included as Electronic Supplementary Material Table S4). A topographic analogy of the data would be a flat mesa top, where severe enough conditions give similar results. Several conditions (60 min, 85 °C; 54 min 95 °C; 39 min, 85 °C; 39 min, 99 °C; and 25 min, 95 °C) gave equivalent FAME conversion yield for all species in our experiments. In fact, the data shown in Figure 3 illustrate the effect of varying the conditions in the experimental design for all 3 species investigated. An analysis of variance between the samples of conditions of ≥60 min and ≥75 °C indicated that there were no significant differences in the FAME values obtained for all four biomass samples investigated. This is consistent with a flat 3D linear correlation that is observed when connecting the respective data (as shown by the black plane on the graphs in Fig. 3). Detailed ANOVA did not show any significant influence of time and temperature on the overall FAME yield for the points captured by the black plane in Fig. 3. These observations suggest that our HCl/MeOH procedure is robust around experimental conditions of 85 °C and 60 min incubation. Furthermore, a historical study of method precision on 26 individual measurements of the *Nannochloropsis* sp. (our reference material), over 5 days and between two analysts, a relative standard deviation of 1.95% with a total FAME content of 10.38 ± 0.20% DW.

### Tolerance of the reaction to water in algal biomass

To investigate how the presence of water in the biomass samples affects the overall FAME yield, we spiked dried biomass with water up to 80% water, representing a 20% solids biomass sample, similar to the product obtained after harvest by centrifugation. The results as averaged FAME yields are shown in Table 3. For both replete biomass samples (*C. vulgaris* replete and *Nannochloropsis*) investigated, the presence of water in algal biomass did not change the overall FAME recovery up to a moisture level of 50%. For both replete biomass samples, the moisture level could increase up to 80% without affecting the recovery of individual fatty acids (data not shown). However, for one sample, deplete *C. vulgaris*, a significant drop in overall FAME yield and an associated significant reduction in the precision (24% drop in yield compared with the reference dry sample and the precision of the measurement dropped 10-fold from ~1% RSD to over 10%) was observed when the reaction was carried out at 80% moisture. Several hypotheses could explain the occurrence of this behavior specifically for the deplete *C. vulgaris* sample; (a) the solubility of the non-polar (high triglyceride) lipids in a highly polar reaction medium (acidic methanol) could compromise the reaction efficiency by limiting access of the catalyst to the substrate, (b) the higher water content reduces the overall catalyst concentration that may be needed for conversion of relatively high concentrations of non-polar lipids, and (c) access of the catalyst to the lipids through inadequate penetration of the catalyst through the algal cell wall, which is thought to be more recalcitrant in deplete algal cells. This difference in algal cell wall structure and recalcitrance can explain why the effect of reduced yield and precision was observed for the deplete *C. vulgaris* sample and not for the replete *C. vulgaris* or *Nannochloropsis* sp. samples, where the overall lipid composition is much more polar and would allow for easier penetration of the solvent and catalyst in the biomass (even in the presence of large amounts of water).

**Table 3:** Summary of conversion efficiency of FAME yield in the presence of increasing levels of water (0–80% moisture or 100–20% solids) on the efficiency of conversion of *in situ* transesterification with the HCl/MeOH method for *Nannochloropsis* sp., *C. vulgaris* deplete, and *C. vulgaris* replete

	Solids (%)	FAME (% DW)
*Nannochloropsis* sp.	100	10.43 ± 0.09
	89.44 ± 0.57	10.38 ± 0.21
	69.75 ± 0.58	10.26 ± 0.15
	50.49 ± 0.65	10.37 ± 0.07
	19.76 ± 0.1	10.12 ± 0.29
*C. vulgaris* deplete	100	56.32 ± 0.46
	89.3 ± 2.41	56.55 ± 0.78
	69.41 ± 1.47	55.55 ± 0.54
	50 ± 0	56.79 ± 1.05
	21.33 ± 0.17	43.05 ± 5.99
*C. vulgaris* replete	100	9.31 ± 0.07
	90 ± 1.32	9.16 ± 0.15
	70.85 ± 5.06	9.17 ± 0.13
	50.22 ± 0.72	9.36 ± 0.12
	20.31 ± 0.29	9.01 ± 0.06

Each measurement was performed in triplicate with values representing mean of the measurements ± SD and show the FAME yield (% DW) expressed on the basis of the original dry weight (or 100% solids)

### The FAME mass balance around an extraction process

To demonstrate the applicability of this one-stage *in situ* transesterification procedure in combination with an extraction-based process, we measured the FAME mass balance around a lipid extraction procedure, that is; the FAME content of the whole biomass, the extract and the residual biomass. This type of measurement is useful and perhaps necessary to illustrate the completeness of a certain extraction procedure. We show that with the HCl/MeOH procedure we can account for all fatty acids in an extraction procedure, i.e., closing the FAME mass balance to within 100 ± 5% (Table 4). The data in Table 4 also show that the Soxhlet lipid extraction procedure, in the case of *Nannochloropsis* sp. biomass extracts 94.3% of the fatty acids of the whole biomass sample, however, for both the *C. vulgaris* biomass samples, deplete and replete, the extraction procedure only extracts 34.1 and 44.5% of the fatty acids respectively. This emphasizes that our procedure is applicable to lipid extracts and residual biomass alike and allows for the reporting of completeness of extraction (as the fraction of fatty acids extracted relative to the fraction in the whole biomass) when performing a lipid extraction procedure.

**Table 4:** Summary of FAME mass balance around a Soxhlet lipid extraction for for *Nannochloropsis* sp., *C. vulgaris* deplete, and *C. vulgaris* replete (without a NaCl wash of the extract)

	Whole biomass	Extract	Residue	Extract + residue	Recovery
*Nannochloropsis* sp.	10.41 ± 0.24	9.82 ± 0.5	0.6 ± 0.05	10.42 ± 0.49	100.06 ± 3.48
*C. vulgaris* deplete	55.54 ± 0.78	18.93 ± 0.22	38.08 ± 2.2	57.01 ± 2.08	102.67 ± 3.87
*C. vulgaris* replete	9.38 ± 0.36	4.17 ± 0.11	4.75 ± 0.01	8.92 ± 0.12	95.19 ± 2.41

Lipids were extracted by Soxhlet and total FAMEs were determined in whole biomass, extract, and residue using the same HCl/MeOH *in situ* procedure. Data shown are FAME yields (% DW of whole biomass) shown as the mean ± SD of triplicate measurements and expressed. Recovery is the sum of the FAMEs in the extract + the FAMEs in the residue and expressed as a fraction of the FAMEs in the whole biomass

## Conclusions

Lipid extraction based on gravimetric solvent recovery is inherently variable as well as inaccurate due to the extraction of non-fatty acid based compounds such as proteins and pigments, making the quantitative determination of lipids in algal biomass very difficult. In this paper, we report on the applicability of a small-scale, single step, acid-catalyzed procedure for quantitative determination of algal lipids as FAMEs through *in situ* whole biomass transesterification.

We demonstrate that a single step procedure based on *in situ* HCl/MeOH catalysis of algal biomass is applicable to small quantities of biomass and the obtained yields are comparable to the AOAC 991.39 two-stage NaOMe/BF_3_ procedure. The reagents for the HCl/MeOH procedure are much easier to prepare, more stable and thus the procedure can more readily be adopted by other researchers. For biomass quantities of between 4 and 7 mg of biomass, we have shown a precision of 1.95% relative standard deviation on 26 independent measurements.

We present data on the efficiency of transesterification of different catalysts at varying temperature, time and pretreatment conditions, which allowed for the statistical analysis of effects. The main metric for the efficiency of the transesterification reaction is the total FAME yield as well as the distribution or profile of the individual fatty acids. When varying reaction conditions around 85 °C and 60 min we found no significant differences in total FAME yield, suggesting that for the strains we investigated, the HCl-catalyzed method has proven to be robust over a wide range of conditions. The small-scale and high precision of the method could allow for automation of the procedure and adaptation to a robotics platform. Work in this area is warranted because of the large number of samples that are typically generated in an algal research or production process.

The FAME yield did not change significantly when the moisture content of the biomass was increased to 50%. At higher levels of moisture in the biomass, there was a significant drop in FAME yield for deplete *C. vulgaris* only. This suggests a high tolerance of the reaction to water in the biomass, which makes the procedure applicable to a range of different biomass samples and greatly simplifies the procedure.

Finally, we demonstrate that we can close the total fatty acid balance around an extraction procedure, illustrating the applicability of this method in conjunction with a lipid extraction process to measure the overall lipid extraction yield, and completeness relative to the total amount of fatty acids present in the biomass.

## Electronic supplementary material

Below is the link to the electronic supplementary material.

ESM 1 (PDF 894 kb)
