# Supplementary material for: Accurate and reliable quantification of total microalgal fuel potential as fatty acid methyl esters by in situ transesterification
Source: Anal Bioanal Chem. 2012 Feb 18;403(1):167–78. doi: 10.1007/s00216-012-5814-0 (PMC3309134; doi:10.1007/s00216-012-5814-0)
Supplement: Supplementary file 1 — (PDF 894 kb) [file 216_2012_5814_MOESM1_ESM.pdf]

Analytical and Bioanalytical Chemistry

Electronic Supplementary Material

Accurate and reliable quantification of total microalgal fuel potential as fatty acid methyl esters by in situ transesterification

Lieve M.L. Laurens, Matthew Quinn, Stefanie Van Wychen, David W. Templeton,

Edward J. Wolfrum

Text S1:Analysis script

```
 # Scripts used for statistical analysis of the results presented in
# "Accurate and reliable quantification of total microalgal fuel potential as fatty acid methyl esters by in situ transesterification"
# R version 2.13.1 (2011-07-08) http://www.R-project.org
# by Lieve Laurens - November 2011
# National Renewable Energy Laboratory

setwd("~/Documents/In situ transesterification")

library(gplots)

# Figure 1: Quantitative extracted lipid yields of Nannochloropsis sp. biomass

ext <- read.csv(file = "Extractions.csv", header = T, stringsAsFactors = FALSE)
ext.mean <- aggregate(ext[,c("extract")],list(ext$methods),mean)
ext.sd <- aggregate(ext[,c("extract")],list(ext$methods),sd)
ci.l <- ext.mean[,2] + ext.sd[,2]
ci.u <- ext.mean[,2] - ext.sd[,2]
barplot2(ext.mean[,2], names.arg = ext.mean[,1], plot.grid = T , plot.ci = TRUE, ci.l = ci.l, ci.u = ci.u, ylab = "Extract (% DW)")

#statistics
j <- aov(ext[,c("extract")] ~ factor(ext$methods)); TukeyHSD(j)



# Figure 2: FAME content using 9 different conditions (catalysts, varying reaction conditions, ...)

cat <- read.csv(file = "Catalyst.csv", header = TRUE, stringsAsFactors = FALSE)
cat.mean <- aggregate(cat[,c("FAME")],list(cat$X),mean)
cat.sd <- aggregate(cat[,c("FAME")],list(cat$X),sd)
ci.l <- cat.mean[,2] + cat.sd[,2]
ci.u <- cat.mean[,2] - cat.sd[,2]

par(las = 3, mar = c(7,4,4,2)+0.1)
barplot2(cat.mean[,2], names.arg = cat.mean[,1], plot.grid = T , plot.ci = TRUE, ci.l = ci.l, ci.u = ci.u, ylab = "FAME (% DW)")
text(locator(1),"ND")

# statistics
j <- aov(cat[,c("FAME")] ~ factor(cat$X)); TukeyHSD(j)

# Figure 3: FAME yield as a function of time and temperature for 4 species
timeTemp <- read.csv(file = "TimeTemp.csv", header = TRUE, stringsAsFactors = FALSE)

library(scatterplot3d)
```

```
plotExpDesign <-  function(alga) {

	x <- timeTemp[which(timeTemp$Strain == alga),"time"]
	y <- timeTemp[which(timeTemp$Strain == alga),"temp"]
	z <- timeTemp[which(timeTemp$Strain == alga),"FAME"]

	s3d <- scatterplot3d(x, y, z, highlight.3d=TRUE,col.axis="black",
	  col.grid="lightblue", main= alga, pch=8, xlab = "Time (min)", ylab = "Temp (ºC)",
zlab = "FAME (%DW)")
	my.lm <- lm(timeTemp[which(timeTemp$Strain == alga),"FAME"] ~
timeTemp[which(timeTemp$Strain == alga),"time"] + timeTemp[which(timeTemp$Strain ==
alga),"temp"])
print(	summary(my.lm))
	s3d$plane3d(my.lm, lty = "dotted", col = "black")
	my.lm <- lm(timeTemp[which(timeTemp$Strain == alga & timeTemp$time > 30 &
timeTemp$temp > 75),"FAME"] ~ timeTemp[which(timeTemp$Strain == alga &
timeTemp$time > 30 & timeTemp$temp > 75),"time"] + timeTemp[which(timeTemp$Strain
== alga & timeTemp$time > 30 & timeTemp$temp > 75),"temp"])
	print(	summary(my.lm))
	s3d$plane3d(my.lm, lty = "solid")

}

quartz(height = 12, width = 12)
par(mfrow = c(2,2))


alga <- "Cv.Replete"
plotExpDesign(alga=alga)
alga <- "Cv.Deplete"
plotExpDesign(alga = alga)
alga <- "Nanno"
plotExpDesign(alga = alga)
alga <- "Phaeo"
plotExpDesign(alga = alga)


#statistics

# analysis of variance of the FAME content with varying time and temperature
j <- aov(FAME ~ time+temp, data= timeTemp[which(timeTemp$Strain == "Cv.Replete"),]);
summary(j)
j <- aov(FAME ~ time+temp, data= timeTemp[which(timeTemp$Strain == "Cv.Deplete"),]);
summary(j)
j <- aov(FAME ~ time+temp, data= timeTemp[which(timeTemp$Strain == "Nanno"),]);
summary(j)
j <- aov(FAME ~ time+temp, data= timeTemp[which(timeTemp$Strain == "Phaeo"),]);
summary(j)

# show that above a certain temperature the differences become insignificant above 75C
```

```
j <- aov(FAME ~ time+temp, data= timeTemp[which(timeTemp$Strain == "Cv.Replete" &
timeTemp$temp > 75),]); summary(j)
j <- aov(FAME ~ time+temp, data= timeTemp[which(timeTemp$Strain == "Cv.Deplete" &
timeTemp$temp > 75),]); summary(j)
j <- aov(FAME ~ time+temp, data= timeTemp[which(timeTemp$Strain == "Nanno" &
timeTemp$temp > 75),]); summary(j)
j <- aov(FAME ~ time+temp, data= timeTemp[which(timeTemp$Strain == "Phaeo" &
timeTemp$temp > 75),]); summary(j)

# show that above a certain time the differences become insignificant at times longer than 30
min
j <- aov(FAME ~ time+temp, data= timeTemp[which(timeTemp$Strain == "Cv.Replete" &
timeTemp$time > 30),]); summary(j)
j <- aov(FAME ~ time+temp, data= timeTemp[which(timeTemp$Strain == "Cv.Deplete" &
timeTemp$time > 30),]); summary(j)
j <- aov(FAME ~ time+temp, data= timeTemp[which(timeTemp$Strain == "Nanno" &
timeTemp$time > 30),]); summary(j)
j <- aov(FAME ~ time+temp, data= timeTemp[which(timeTemp$Strain == "Phaeo" &
timeTemp$time > 30),]); summary(j)

# show that above a certain time/temp the differences become insignificant >75C and > 30
min
j <- aov(FAME ~ time+temp, data= timeTemp[which(timeTemp$Strain == "Cv.Replete" &
timeTemp$time > 30 & timeTemp$temp > 75),]); summary(j)
j <- aov(FAME ~ time+temp, data= timeTemp[which(timeTemp$Strain == "Cv.Deplete" &
timeTemp$time > 30 & timeTemp$temp > 75),]); summary(j)
j <- aov(FAME ~ time+temp, data= timeTemp[which(timeTemp$Strain == "Nanno" &
timeTemp$time > 30 & timeTemp$temp > 75),]); summary(j)
j <- aov(FAME ~ time+temp, data= timeTemp[which(timeTemp$Strain == "Phaeo" &
timeTemp$time > 30 & timeTemp$temp > 75),]); summary(j)




#####################################################
### END OF FILE ### END OF FILE ### END OF FILE ###
```

Table S1: Raw data for extraction variability presented in Figure 1

methods	method.2	extract
soxhlet.1	soxhlet.16h	31,04
soxhlet.1	soxhlet.16h	30,71
soxhlet.1	soxhlet.16h	30,34
soxhlet.1	soxhlet.16h	32,15
soxhlet.2	soxhlet.3h.w	23.283
soxhlet.2	soxhlet.3h.w	23.046
soxhlet.2	soxhlet.3h.w	21.551
ASE.1	ASE.40C.500p	33.601
ASE.1	ASE.40C.500p	33.939
ASE.1	ASE.40C.500p	33.980
ASE.2	ASE.100C.2000p	39,08
ASE.2	ASE.100C.2000p	39,07
ASE.2	ASE.100C.2000p	38,68
ASE.3	ASE.40C.500p.H	7,07
ASE.3	ASE.40C.500p.H	7,44
ASE.3	ASE.40C.500p.H	7,12
ASE.4	ASE.100C.2000p.H	17,79
ASE.4	ASE.100C.2000p.H	18,13
ASE.4	ASE.100C.2000p.H	17,74

Table S2: Raw data for catalyst study reported in Figure 2

	Catalyst	strain	type	time	FAME
NaOMe.BF3.1	AOAC.991.39	Nannochloropsis	acid.base	35	10,3661698
NaOMe.BF3.1	AOAC.991.39	Nannochloropsis	acid.base	35	10,6448669
NaOMe.BF3.1	AOAC.991.39	Nannochloropsis	acid.base	35	10,5304166
NaOMe.BF3.1	AOAC.991.39	Nannochloropsis	acid.base	35	10,89
NaOMe.BF3.1	AOAC.991.39	Nannochloropsis	acid.base	35	10,73
NaOMe.BF3.1	AOAC.991.39	Nannochloropsis	acid.base	35	10,84
NaOMe.BF3.1	AOAC.991.39	Nannochloropsis	acid.base	35	10,6150179
NaOMe.BF3.1	AOAC.991.39	Nannochloropsis	acid.base	35	10,2841782
NaOMe.BF3.1	AOAC.991.39	Nannochloropsis	acid.base	35	10,5312541
NaOMe.BF3.1	AOAC.991.39	Nannochloropsis	acid.base	35	10,1391647
NaOMe.BF3.1	AOAC.991.39	Nannochloropsis	acid.base	35	10,2341877
NaOMe.BF3.1	AOAC.991.39	Nannochloropsis	acid.base	35	10,5674038
HCl.MeOH.1	NREL.HCl	Nannochloropsis	acid	60	10,3400016
HCl.MeOH.1	NREL.HCl	Nannochloropsis	acid	60	10,6096221
HCl.MeOH.1	NREL.HCl	Nannochloropsis	acid	60	10,3279988
HCl.MeOH.1	NREL.HCl	Nannochloropsis	acid	60	10,4632
HCl.MeOH.1	NREL.HCl	Nannochloropsis	acid	60	10,4992156
TMG	TMG	Nannochloropsis	base	5	6,96
TMG	TMG	Nannochloropsis	base	5	7,64
TMG	TMG	Nannochloropsis	base	5	8,34
TMG	TMG	Nannochloropsis	base	5	7,17
TMG	TMG	Nannochloropsis	base	5	7,08
TMG	TMG	Nannochloropsis	base	5	7,17
TMG	TMG	Nannochloropsis	base	5	8,81
TMG	TMG	Nannochloropsis	base	5	8,26
TMG	TMG	Nannochloropsis	base	5	8,2

NaOMe	NaOMe	Nannochloropsis	base	5	0
NaOMe	NaOMe	Nannochloropsis	base	5	0
NaOMe	NaOMe	Nannochloropsis	base	5	0
HCl.MeOH.1	NREL.HCl	Nannochloropsis	acid	60	10,79
HCl.MeOH.1	NREL.HCl	Nannochloropsis	acid	60	10,53
HCl.MeOH.1	NREL.HCl	Nannochloropsis	acid	60	10,6351361
HCl.MeOH.1	NREL.HCl	Nannochloropsis	acid	60	10,4195698
HCl.MeOH.1	NREL.HCl	Nannochloropsis	acid	60	10,5792685
HCl.MeOH.1	NREL.HCl	Nannochloropsis	acid	60	10,5097181
HCl.MeOH.1	NREL.HCl	Nannochloropsis	acid	60	10,381
HCl.MeOH.1	NREL.HCl	Nannochloropsis	acid	60	10,617
HCl.MeOH.1	NREL.HCl	Nannochloropsis	acid	60	10,49
HCl.MeOH.1	NREL.HCl	Nannochloropsis	acid	60	12,722
HCl.MeOH.1	NREL.HCl	Nannochloropsis	acid	60	10,744
HCl.MeOH.1	NREL.HCl	Nannochloropsis	acid	60	10,428
HCl.MeOH.1	NREL.HCl	Nannochloropsis	acid	60	10,409
HCl.MeOH.1	NREL.HCl	Nannochloropsis	acid	60	10,374
HCl.MeOH.1	NREL.HCl	Nannochloropsis	acid	60	10,922
HCl.MeOH.1	NREL.HCl	Nannochloropsis	acid	60	10,73
HCl.MeOH.1	NREL.HCl	Nannochloropsis	acid	60	10,788
HCl.MeOH.2	NREL.HCl.noCM	Nannochloropsis	acid	60	10,3573287
HCl.MeOH.2	NREL.HCl.noCM	Nannochloropsis	acid	60	10,4514842
HCl.MeOH.2	NREL.HCl.noCM	Nannochloropsis	acid	60	10,6543872
NaOMe.BF3.2	micro.991.39	Nannochloropsis	acid.base	35	10,6017737
NaOMe.BF3.2	micro.991.39	Nannochloropsis	acid.base	35	10,6354279
NaOMe.BF3.2	micro.991.39	Nannochloropsis	acid.base	35	10,7642059
NaOMe.BF3.2	micro.991.39	Nannochloropsis	acid.base	35	10,23
NaOMe.BF3.2	micro.991.39	Nannochloropsis	acid.base	35	10,57
NaOMe.BF3.2	micro.991.39	Nannochloropsis	acid.base	35	10,14

BF3	BF3	Nannochloropsis	acid	35	9,74927967
BF3	BF3	Nannochloropsis	acid	35	9,97254402
BF3	BF3	Nannochloropsis	acid	35	9,81523606
mAOAC.922	AOAC.922	Nannochloropsis	base.acid		6,64
mAOAC.922	AOAC.922	Nannochloropsis	base.acid		6,31
mAOAC.922	AOAC.922	Nannochloropsis	base.acid		7,46
mAOAC.989	AOAC.989	Nannochloropsis	acid		3,78
mAOAC.989	AOAC.989	Nannochloropsis	acid		3,5
mAOAC.989	AOAC.989	Nannochloropsis	acid		3,08

Table S3: Raw data for time and temperature interactions on total FAME response (based on experimental design)

Strain	time	temp	FAME
Cv.Deplete	39,5	71	9,35138347
Cv.Deplete	39,5	71	9,22642521
Cv.Deplete	25	75	8,69333715
Cv.Deplete	25	75	8,79711429
Cv.Deplete	54	75	9,83840289
Cv.Deplete	54	75	9,39530271
Cv.Deplete	19	85	9,26264538
Cv.Deplete	19	85	9,24135346
Cv.Deplete	60	85	9,72127272
Cv.Deplete	60	85	9,58250441
Cv.Deplete	39,5	85	9,70326219
Cv.Deplete	39,5	85	9,40540267
Cv.Deplete	39,5	85	9,59706773
Cv.Deplete	39,5	85	9,57450699
Cv.Deplete	39,5	85	9,50177447
Cv.Deplete	25	95	10,0692024
Cv.Deplete	25	95	10,005994
Cv.Deplete	54	95	9,47911475
Cv.Deplete	54	95	9,51889956
Cv.Deplete	39,5	99,14	9,59235971
Cv.Deplete	39,5	99,14	9,67888257
Cv.Replete	39,5	71	44,1499047
Cv.Replete	39,5	71	41,7176853
Cv.Replete	25	75	38,5464441
Cv.Replete	25	75	43,3060809
Cv.Replete	54	75	54,4678852

Cv.Replete	54	75	51,9676452
Cv.Replete	19	85	48,3895128
Cv.Replete	19	85	49,7678182
Cv.Replete	60	85	60,697067
Cv.Replete	60	85	59,6912157
Cv.Replete	39,5	85	59,3374208
Cv.Replete	39,5	85	57,8574368
Cv.Replete	39,5	85	57,6898667
Cv.Replete	39,5	85	59,0413861
Cv.Replete	39,5	85	59,1132766
Cv.Replete	25	95	61,8371763
Cv.Replete	25	95	63,5469955
Cv.Replete	54	95	60,7659138
Cv.Replete	54	95	62,9681751
Cv.Replete	39,5	99,14	60,3632555
Cv.Replete	39,5	99,14	60,006168
Nanno	39,5	71	10,7451868
Nanno	39,5	71	10,6392304
Nanno	25	75	10,1560581
Nanno	25	75	10,619519
Nanno	54	75	10,5270283
Nanno	54	75	10,541288
Nanno	19	85	10,5321546
Nanno	19	85	10,4098023
Nanno	60	85	11,0137448
Nanno	60	85	11,123797
Nanno	39,5	85	10,8349856
Nanno	39,5	85	10,8744358
Nanno	39,5	85	11,189628

Nanno	39,5	85	11,009422
Nanno	25	95	10,6890734
Nanno	25	95	10,5048117
Nanno	54	95	10,9444261
Nanno	54	95	10,8446819
Nanno	39,5	99,14	10,8694398
Nanno	39,5	99,14	10,8807655
Phaeo	39,5	71	7,32013324
Phaeo	39,5	71	6,86990175
Phaeo	25	75	7,34887284
Phaeo	25	75	7,19967903
Phaeo	54	75	7,8022632
Phaeo	54	75	7,02306652
Phaeo	19	85	7,87946668
Phaeo	19	85	7,81281528
Phaeo	60	85	7,79226307
Phaeo	60	85	7,72755843
Phaeo	39,5	85	7,91216034
Phaeo	39,5	85	7,92111971
Phaeo	39,5	85	8,01755129
Phaeo	39,5	85	8,08951455
Phaeo	39,5	85	8,36488877
Phaeo	25	95	7,9455125
Phaeo	25	95	7,64875514
Phaeo	54	95	8,34505977
Phaeo	54	95	7,80337307
Phaeo	39,5	99,14	8,13147606
Phaeo	39,5	99,14	7,68850135

Table S4: Analysis of variance table (partial sum of squares - Type III) for Response Surface Reduced Quadratic Model

	**Nannochloropsis**					**Phaeodactylum**				
Source	Sum of Squares	df	Mean Square	F Value	p-value Prob > F	Sum of Squares	df	Mean Square	F Value	p-value Prob > F
Model	0,85	4	0,21	7,57	0.0015	2,22	4	0,56	9,57	0.0004
A-Time	0,42	1	0,42	14,78	0.0016	0,02	1	0,02	0,37	0.5510
B-Temp	0,17	1	0,17	6,10	0.0260	1,37	1	1,37	23,52	0.0002
A^2	0,20	1	0,20	7,02	0.0182	0,18	1	0,18	3,11	0.0968
B^2	0,18	1	0,18	6,38	0.0233	0,82	1	0,82	14,20	0.0017
Residual	0,42	15	0,03			0,93	16	0,06		
Lack of Fit	0,20	4	0,05	2,39	0.1140	0,08	4	0,02	0,29	0.8766
Pure Error	0,23	11	0,02			0,85	12	0,07		
Cor Total	1,27	19				3,15	20			

	**replete C. vulgaris**					**deplete C. vulgaris**				
Source	Sum of Squares	df	Mean Square	F Value	p-value Prob > F	Sum of Squares	df	Mean Square	F Value	p-value Prob > F
Model	1,89	3	0,63	31,46	< 0.0001	1021,87	3	340,62	37,95	< 0.0001
A-Time	0,20	1	0,20	10,08	0.0055	184,77	1	184,77	20,59	0.0003
B-Temp	0,69	1	0,69	34,62	< 0.0001	751,08	1	751,08	83,68	< 0.0001
AB	0,99	1	0,99	49,68	< 0.0001	86,02	1	86,02	9,58	0.0066
Residual	0,34	17	0,02			152,58	17	8,98		
Lack of Fit	0,16	5	0,03	2,21	0.1207	127,38	5	25,48	12,13	0.0002
Pure Error	0,18	12	0,01			25,20	12	2,10		
Cor Total	2,23	20				1174,45	20			
